# Spare Hours1Paper read before the annual meeting of the Pennsylvania State Veterinary Medical Association, March 6, 1901.

**Published:** 1901-07

**Authors:** B. M. Underhill

**Affiliations:** Philadelphia


### SPARE HOURS.[1]

By B. M. Underhill,
PHILADELPHIA.

Some two years ago, while travelling in the West, and happening near a town where a young veterinarian was located in whom I had a friendly interest, I went there to call upon him. He was

[1] Paper read before the annual meeting of the Pennsylvania State Veterinary Medical Association, March 6, 1901.

not in his office at the time, and, as the general appearance of the latter would not indicate a very prosperous tenant, I made inquiry of a neighboring merchant as to the degree of success he was having. I was told that during the few years he had been located in the town he had made many friends; he seemed to have set a high mark for himself, and had built up quite a profitable practice. But during the past year he had joined a club, and now whenever he came in from his professional duties he lost no time in getting to this resort to engage in the game of chess, at which he had become quite proficient, and it seemed that the time he wished to devote to the game that had so fascinated him was being less and less disturbed by his practice.

This course of events is a familiar narrative to those of us who are observers of human affairs, and no doubt we can all recall one or more cases similar in general, many of them worse in detail.

Failures in professional life are numerous, and it is not the purpose of this paper to deal with their cause nor with their remedy.

But we see examples here and there of a quality of failure among veterinarians that has no reference to their success in winning clients nor to their ability as practitioners—failures that may be accompanied by successful careers from a pecuniary point of view, and yet failures when viewed from the higher standard. If the veterinarian who is well-known in a community sees fit to spend his evenings in a pool-room or plays euchre at the livery stable office during spare hours in the day, he may do so without affecting his clientage in the least, especially if his reputation for professional skill is well established. Should his brother in medicine occupy his leisure with such pastimes it would cost him the confidence of his clients. He would be counted a trifler who valued his time lightly and whose tastes were not in keeping with the dignity of his profession. Communities, as a rule, hold the calling of the medical man in high esteem, and any deportment unbecoming to it is readily noticed. Their ideas of the profession as a whole are influenced by the impression that its, to them, most familiar representative creates, and the respect that any profession may claim must be created for it by the regard that each individual member of that profession may establish for himself. If every qualified veterinarian were ashamed to waste his spare time at trifles or to be seen in association with idle men, then would the profession be placed upon a still higher pedestal and a higher standard of individual deportment be expected of its members. Locate in whatever town or neighborhood in a large city you may, you will find that the estimate of the veterinary profession in that community is gauged almost absolutely by the character of the individual representing it previous to your coming. If he was a man who commanded respect his profession is likewise respected, and more will be expected of you than would be the case were your predecessor a man of careless habits.

But, no matter how busy the veterinarian may be, he must have hours of relaxation, and most all who are engaged in a general practice will have periods when half days or whole days will come and go without a demand for their services. This is especially true of the young practitioner recently located and in the midst of a field of work that he must attract to himself through proof of his worthiness for it. If he enters upon a practice in a locality that is strange to him he will probably find that the first ones to seek his acquaintance and to make plausible assertions of a friendly interest in his welfare will be the idle young men about town, and often the feeling of loneliness and weary hours of waiting for practice may strongly tempt the young veterinarian to partake of associations and pastimes that, under other circumstances, would repel rather than attract him. On the other hand, the busy, substantial citizens may show an indifference toward him, or manifest by their manner that they are absolutely oblivious to his presence until he shall prove to them that he is a useful and worthy acquisition to the community.

Perhaps this is a subject that should be addressed more particularly to the undergraduate or to the beginner in practice, whose experience has not yet revealed to him the reefs he must steer clear of in his progress toward a realization of his ideals. But those riper in experience may well give a thought to the husbanding of time not in demand by professional duties—a thought directed toward progress and the higher attributes of their calling.

However, as the child at school must have his hour of recess that he may break away from the restraint of the class-room to return refreshed and fortified for his work, so and for the same reason must the busy man have his half-day to go fishing, his brush on the speedway, or his evening at the theatre. This is nature's demand, and to disregard it invites a severe penalty. Then, again, time will be taken by the conscientious veterinarian for technical reading. He must read up his cases and keep in touch with the progress of his profession through careful perusal of one or more of the periodicals devoted to it. But time so used cannot be considered as leisure. It is time taken for a part of his work that a due regard for his clients, as well as himself, bids him not to neglect.

It is the inevitable period of slackness in demand upon the veterinarian's service—the hour or the day forced upon his disposal—that is so often allowed to pass as so much time lost without a thought of the opportunity that it offers to partake of intellectual nourishment, an opportunity which, if accepted and used wisely, will establish a habit of study, a refinement of taste, and a happy broadening view of life's purpose that the learning of the specialist in any particular department of science is inadequate to afford. An escape now and then from the cold calculations of science for an hour's interview with the classics lends a seasoning to the plainest of lives and makes one good company for himself amid surroundings that are perhaps not always congenial. It generates habits of good reading and high thinking, opening up new fields of such fascinating interest that the hour devoted to them becomes one of pleasure.

By thus utilizing such time as may be spared we who have not had the advantage that a college education affords can know something of the college outlook—a prospect that at least has the merit of time redeemed in its attainment.

